# Integrated Cytological, Physiological, and Comparative Transcriptome Profiling Analysis of the Male Sterility Mechanism of ‘Xinli No.7’ *Pear* (*Pyrus* sp.)

**DOI:** 10.3390/plants14121783

**Published:** 2025-06-11

**Authors:** Hao Li, Xiangyü Li, Yüjia Luo, Quanhui Ma, Zhi Luo, Jiayuan Xuan, Cuiyun Wu, Fenfen Yan

**Affiliations:** 1 The National and Local Joint Engineering Laboratory of High Efficiency and Superior-Quality Cultivation and Fruit Deep Processing Technology of Characteristic Fruit Trees, College of Horticulture and Forestry, Tarim University, Alar 843300, China; lihao2878@163.com (H.L.); lxyzky@taru.edu.cn (X.L.); lluoyujia@163.com (Y.L.); ma.qui@163.com (Q.M.); lz15930225993@126.com (Z.L.); x18294881852@163.com (J.X.); wcyby@163.com (C.W.); 2 Xinjiang Production and Construction Crops Key Laboratory of Protection and Utilization of Biological Resources in Tarim Basin, Tarim University, Alar 843300, China; 3 College of Life Science and Technology, Tarim University, Alar 843300, China

**Keywords:** pear, ‘Xinli No.7’, male sterility, ROS-scavenging systems, transcriptome

## Abstract

*Pyrus bretschneideri* ‘Xinli No.7’, a progeny of *Pyrus sinkiangensis* ‘Korla Fragrant Pear’, is an early-maturing, high-quality pear (*Pyrus* spp.) cultivar. As a dominant variety in China’s pear-producing regions, it holds significant agricultural importance. Investigating its male sterility (MS) mechanisms is critical for hybrid breeding and large-scale cultivation. Integrated cytological, physiological, and transcriptomic analyses were conducted to compare dynamic differences between male sterility (MS, ‘Xinli No.7’) and male-fertile (MF, ‘Korla Fragrant Pear’) plants during anther development. Cytological observations revealed that, compared with ‘Korla Fragrant Pear’, the tapetum of ‘Xinli No.7’ exhibited delayed degradation and abnormal thickening during the uninucleate microspore stage. This pathological alteration compressed the microspores, ultimately leading to their abortion. Physiological assays demonstrated excessive reactive oxygen species (ROS) accumulation, lower proline content, higher malondialdehyde (MDA) levels, and reduced activities of antioxidant enzymes (peroxidase and catalase) in MS plants. Comparative transcriptomics identified 283 co-expressed differentially expressed genes (DEGs). Functional enrichment linked these DEGs to ROS-scavenging pathways: galactose metabolism, ascorbate and aldarate metabolism, arginine and proline metabolism, fatty acid degradation, pyruvate metabolism, and flavonoid biosynthesis. qRT-PCR validated the expression patterns of key DEGs in these pathways. A core transcriptome-mediated MS network was proposed, implicating accelerated ROS generation and dysregulated tapetal programmed cell death. These findings provide theoretical insights into the molecular mechanisms of male sterility in ‘Xinli No.7’, supporting future genetic and breeding applications.

## 1. Introduction

The pear (*Pyrus* spp.) originated in Asia, with China as a primary domestication center. Historical records of pear cultivation in China date from the Shijing (Classic of Poetry, ca. 11th-7th century BCE). *Pyrus bretschneideri* ‘Xinli No.7’, is an early-maturing hybrid cultivar developed by Tarim University in 1985. It was bred by crossing ‘Korla Fragrant Pear’ × ‘Zaosu’ *Pear* and exhibits pollen abortion, which severely compromises its self-pollination capacity [1,2]. This reproductive constraint requires strict planting configurations with synchronized flowering and compatible pollinizer varieties, thus complicating cultivar promotion and large-scale cultivation.

Anther morphogenesis has been a central focus in floral organ development research. Male sterility, a common phenomenon across angiosperms, has been reported in 617 cultivars or interspecific hybrids spanning 320 species (162 genera, 43 families) as reported by Kaul [3,4]. Precise identification of abortion timing, defective tissue localization, and cytopathological features is critical for mechanistic investigation of male sterility. Tan et al. [5] discovered in their study on male sterility in ‘xiaoxiangshui’ *Pear* that during the tetrad stage, developmental abnormalities in the tapetum, vacuolation of the middle layer, and suberization of the septal vascular bundles collectively resulted in nutrient supply disruption. Additionally, investigating the physiological and biochemical traits of male sterility facilitates linking genetic profiles to phenotypic variations, thereby clarifying its underlying mechanisms. Wang et al. [6] found that the bud abortion of Brassica napus may be related to the decrease in antioxidant enzyme activities (such as SOD, POD, and CAT). Zhou et al. [7] showed that the abortion of maize (*Zea mays* L.) K932MS may be due to the decrease in CAT and SOD activity and the increase in POD activity in sterile anthers, which are not conducive to the normal growth and development of anthers. In sterile soybean lines, research has shown that the lack of enzymatic and non-enzymatic ROS-scavenging systems leads to high ROS levels and excessive POD, leading to pollen abortion [8]. Breaking the balance of antioxidant systems can result in the abnormal development of anthers in MS wheat [9].

High-throughput sequencing and RNA sequencing (RNA-seq) are powerful tools for profiling the gene expression levels, candidate genes, biosynthetic pathways, and key enzymes involved in MS [10,11]. Dai et al. [12] conducted high-throughput transcriptome sequencing on anther tissues of the sterile male line, revealing that abnormal development of male organs exhibited significant dynamic fluctuations in the expression levels of key genes. These genes are involved in different metabolic processes, such as ROS metabolism [10,13,14], energy metabolism [15], lipid metabolism [15], carbohydrate metabolism [15,16], and signal transduction [17]. Fang et al. [18] speculated that ‘Huayou 2’ belongs to the nuclear–cytoplasmic-interaction type of male sterility and found that the regulatory network of a miRNA-PHAS phasiRNA-target may be involved in the process of nuclear–cytoplasmic-interaction male sterility. Zhang et al. [19] mapped the male sterile gene *MSLP* in the nucleus of ‘Qianyang seedless’ ponkan and obtained three effective pieces of gene information. Wu et al. [20] found that the overexpression of a *CsRAD51* gene in ‘Seedless Ougan’ at the microspore mother cell stage, tetrad stage, and mononuclear pollen stage caused its meiosis abnormality, which ultimately affected its fertility.

This study utilized ‘Xinli No.7’ and ‘Korla Fragrant Pear’ as experimental materials. By conducting cytological observations of stamen developmental structures, analyzing physiological and biochemical differences between the two cultivars, and integrating transcriptome sequencing data, we systematically elucidated the molecular mechanisms underlying pollen abortion in ‘Xinli No.7’. These findings provide critical insights for future research leveraging molecular biology and genetic engineering technologies to dissect male sterility mechanisms in pears.

## 2. Results

### 2.1. Morphological and Cytological Comparison

**Figure 1:**
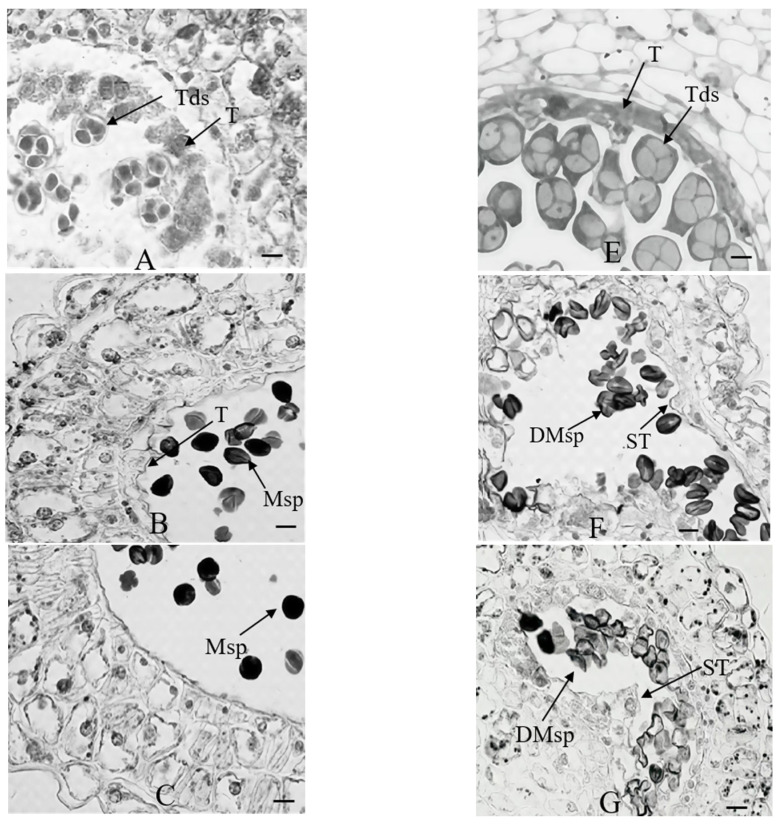

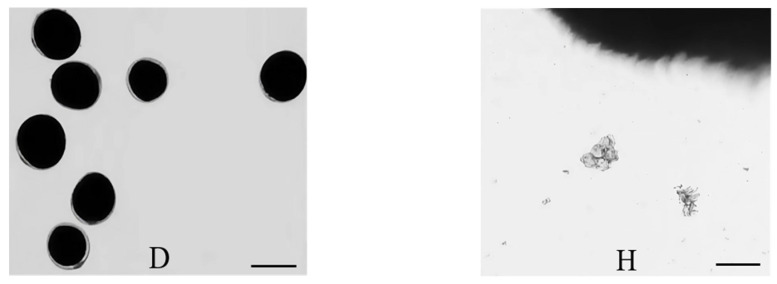
A paraffin section observation of the pollen and anther wall development of ‘Korla Fragrant Pear’ (**left**) and ‘Xinli No.7’ (**right**). ST, swollen tapetum; T, tapetum; Tds, tetrads; MSp, microspores; DMsp, deformed microspore. The scale is 20 μm. (**A**). Following meiosis, the pollen mother cells of ‘Korla Fragrant Pear’ form tetrads that are dispersed within the anther locules. (**B**). During mitotic division of microspores, tapetal cells undergo progressive degeneration to supply essential nutrients for microspore development, while the microspores migrate toward the anther wall. (**C**). At pollen maturity, the tapetum has completely degenerated, and the remaining middle layer cells are compactly arranged to form a smooth inner lining of the anther locules. (**D**). The microspores gradually enlarge and become rounded, with thickening of the exine, ultimately forming spherical pollen grains. (**E**). Following meiosis, the pollen mother cells of ‘Xinli No.7’ form tetrads that are dispersed within the anther locules. ‘Xinli No.7’ tetrad period. (**F**). The tapetal cells neither separate from the middle layer cells nor undergo degeneration or degradation. Instead, they continue to proliferate and gradually encroach into the locule space. (**G**). The tapetum and middle-layer cells undergo excessive abnormal development, resulting in the compression and obliteration of the anther locules, while the microspores undergo degradation. (**H**). ‘Xinli No.7’ pollen vacuolization.

During pollen development, significant differences were observed between ‘Korla Fragrant Pear’ and ‘Xinli No.7’ cultivars. Following meiosis completion, pollen mother cells of ‘Korla Fragrant Pear’ formed tight, callose-encapsulated tetrads (Figure 1A), exhibiting distinct cytoplasmic partitioning between microspores, with a dense cytoplasm devoid of vacuoles. In contrast, ‘Xinli No.7’ exhibited well-developed microsporangium walls, with its tapetum demonstrating cytoplasmic condensation to form an annular structure encircling the sporangium (Figure 1E). In ‘Korla Fragrant Pear’, tetrads released microspores through the action of callase secreted by the tapetum. The liberated microspores subsequently underwent volume expansion and cytoplasmic content accumulation via nutrient absorption from degenerating microsporangium wall cells (Figure 1B). The microspores of ‘Xinli No.7’ displayed aberrant morphology, characterized by indistinct nuclear organization and underdeveloped exine structures, potentially correlated with their pronounced vacuolization phenotype (Figure 1F). In ‘Korla Fragrant Pear’, microspores developed into spherical pollen grains accompanied by programmed cell death (PCD) of the tapetum (Figure 1C). During the uninucleate microspore stage, ‘Xinli No.7’ exhibited delayed degradation and abnormal thickening of the tapetum, which compressed the microspores, causing them to become deformed (Figure 1G). The final I_2_-KI staining revealed that mature pollen grains of ‘Korla Fragrant Pear’ exhibited characteristic deep-blue coloration indicative of starch accumulation (Figure 1D). In ‘Xinli No.7’, aberrant development of the tapetum led to microspore degeneration, leaving only residual structures within the anther locules after dehiscence (Figure 1H).

### 2.2. SOD, POD, CAT, and MDA Activities and Hydrogen Peroxide Content Proline Content and Amino Acid Content

**Figure 2:**
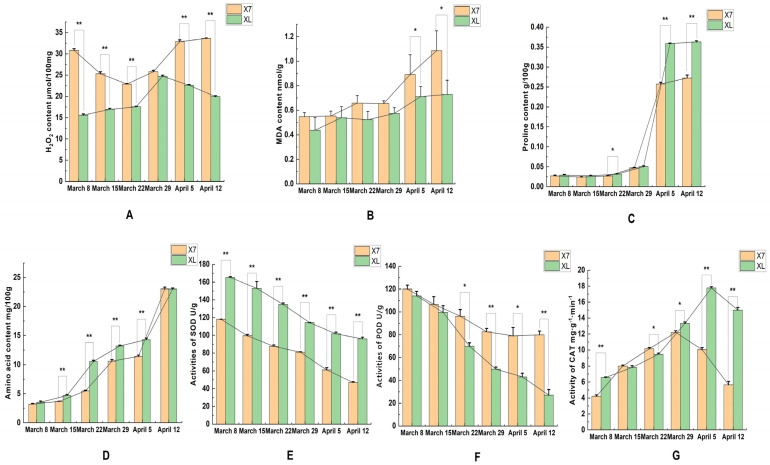
Changes in physiological indexes of anthers of ‘Xinli No.7’ and ‘Korla Fragrant Pear’ in different periods: (**A**). Hydrogen peroxide content (**B**). Malondialdehyde content (**C**). Proline content (**D**). Amino acid content (**E**). SOD activity (**F**). POD activity (**G**). CAT activity. Values are means ± SD of three replicates. Asterisks represent statistically significant differences between ‘Xinli No.7’ and ‘Korla Fragrant Pear’ (Student’s *t*-test * *p* < 0.05, ** *p* < 0.01).

Given that oxidative stress is associated with the excessive production of reactive oxygen species (ROS), and such overproduction can induce structural damage to cellular components, the hydrogen peroxide (H_2_O_2_) content was quantified in ‘Korla Fragrant Pear’ and ‘Xinli No.7’ during the period spanning March 8 to April 12. As shown in Figure 2A, the H_2_O_2_ content in the anthers of ‘Xinli No.7’ was significantly higher than that of ‘Korla Fragrant Pear’ at all time points except for March 29. Although ‘Xinli No.7’ also exhibited higher H_2_O_2_ levels compared to ‘Korla Fragrant Pear’ on March 29, the difference was not statistically significant. These results collectively indicate excessive accumulation of ROS in the anthers of ‘Xinli No.7’.

Malondialdehyde content can reflect the degree of oxidative stress in plants. From Figure 2B, it can be seen that in different periods, the malondialdehyde content of ‘Xinli No.7’ is higher than that of ‘Korla Fragrant Pear’, especially after March 29, the difference between the two is significant. The proline content of flower buds of ‘Korla Fragrant Pear’ and ‘Xinli No.7’ showed a similar trend. Before inflorescence elongation, the free proline content in the flower buds of the two pears increased steadily, and the proline content of ‘Korla Fragrant Pear’ was slightly higher than that of ‘Xinli No.7’ (Figure 2C). Since the beginning of April, the proline content of the anthers of the two varieties increased rapidly, and the content of ‘Korla Fragrant Pear’ was significantly higher than that of ‘Xinli No.7’, which further revealed the close relationship between the male sterility of pear and the difference in proline content. The change trend of total free amino acid content in the two pears was similar, and there was no significant difference on April 12 (Figure 2D).

During the development of flower buds, the activities of SOD and POD in ‘Xinli No.7’ and ‘Korla Fragrant Pear’ showed a decreasing trend. The activities of SOD and POD in ‘Xinli No.7’ were always lower than those in ‘Korla Fragrant Pear’, especially after March 29; the difference was more significant (Figure 2E,F). The CAT activity of ‘Xinli No.7’ and ‘Korla Fragrant Pear’ showed a trend of increasing first and then decreasing during the bud development period, and there was a significant difference between the two after March 29 (Figure 2G).

### 2.3. RNA-Seq Analysis and DEG Comparisons at Different Stages of Anther Development

#### 2.3.1. Evaluation of Sequencing Data of Samples at Different Developmental Stages

This study conducted transcriptome sequencing analysis on 12 samples from ‘Xinli No.7’ and ‘Korla Fragrant Pear’, encompassing both early (X7Q, XLQ) and late (X7H, XLH) stages of pollen abortion. The sequencing analysis revealed that both cultivars across the two developmental stages collectively generated 91.5 Gb of high-quality clean data, as detailed in Table 1. ‘Raw reads’ refers to the total number of entries in the raw sequencing data, ‘Clean bases’ refers to the total amount of sequencing data after quality control, Q20 and Q30 refer to the percentage of bases with sequencing quality above 99% and 99.9% of the total bases, respectively, and ‘GC content (%)’ refers to the percentage of total bases corresponding to the sum of the G and C bases in the quality control data. All sequencing samples met high-quality standards, with Q20 values consistently maintained above 98.9% and Q30 values remaining above 96.4%, confirming the reliability of the sequencing data. Furthermore, the sequencing data demonstrated favorable alignment efficiency with the reference genome, with overall alignment rates ranging from 74.13% to 77.01%. This indicates that the majority of sequencing reads were accurately mapped to the reference genome, establishing a robust foundation for subsequent differential expression analysis.

#### 2.3.2. Correlation Test Between Samples in Different Periods

**Figure 3:**
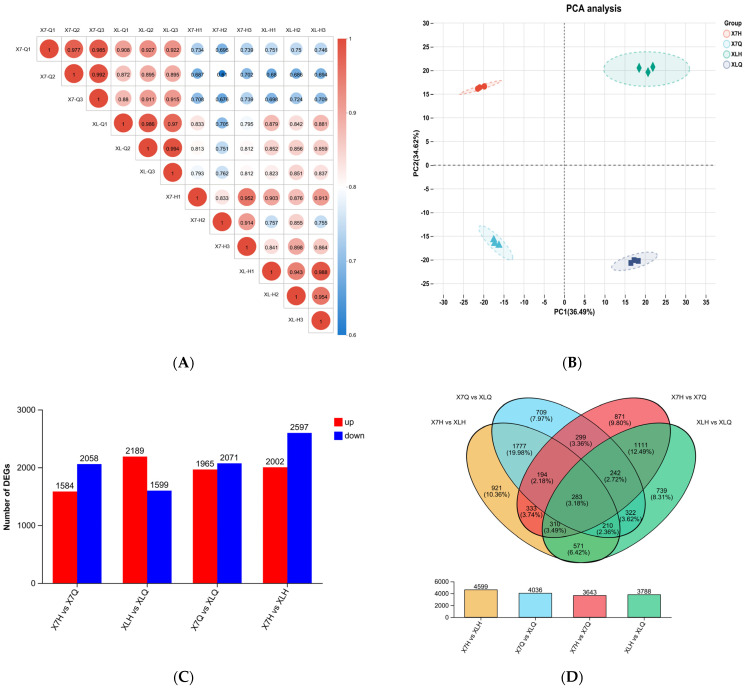
(**A**). Correlation analysis between samples, (**B**). principal component analysis between samples, (**C**). statistical difference in expression in different comparison groups, and (**D**). Wayne diagram of different comparison groups and total number of differential genes in different comparison groups.

Figure 3A is the correlation heat map of the four groups of samples. The results demonstrated that the squared Pearson correlation coefficients (R^2^) among triplicate biological replicates within each experimental group consistently exceeded 0.833, confirming high intra-group reproducibility. Cross-group comparative analysis revealed R^2^ values ranging from 0.872 to 0.927 between X7-Q and XL-Q phases, 0.61 to 0.739 for X7-H phase comparisons, and 0.68 to 0.751 for XL-H phase comparisons, demonstrating significant inter-group disparities between distinct developmental stages. Principal component analysis (PCA) based on FPKM values further confirmed that samples within each group formed tightly clustered groups in the two-dimensional space. This not only validated the rationality of the experimental design but also demonstrated the high reliability of the sample data from an additional perspective.

Figure 3C,D illustrate the comparative results of differentially expressed genes (DEGs) analyzed using DESeq2, with filtering thresholds set at |log_2_FC| ≥ 1 and adjusted *p*-value < 0.05. In the inter-cultivar comparisons, 4599 differentially expressed genes (DEGs) were identified between the X7H and XLH stages, comprising 2002 upregulated and 2597 downregulated genes. Similarly, the comparison of X7Q and XLQ stages revealed 4036 DEGs, with 1965 upregulated and 2071 downregulated genes. In intra-cultivar comparisons across developmental stages, 3643 differentially expressed genes (DEGs) were identified between the X7H and X7Q stages of ‘Xinli No.7’, while 3788 DEGs were detected between the XLH and XLQ stages of ‘Korla Fragrant Pear’. The number of differentially expressed genes (DEGs) between the X7H and XLH phases was significantly higher than that observed between the X7H and X7Q phases. This suggests that physiological and metabolic activities during the late abortion phase are more dynamic and may involve a more complex regulatory network. Further analysis through Venn diagrams of the intersections of differentially expressed genes (DEGs) across the four comparison groups revealed 283 genes that were consistently differentially expressed in all comparisons: X7H vs. XLH, X7Q vs. XLQ, X7H vs. X7Q, and XLH vs. XLQ. These shared differentially expressed genes (DEGs) likely represent conserved core regulatory genes during pear pollen abortion, providing critical candidate gene resources for elucidating the mechanisms underlying abortion.

#### 2.3.3. KEGG Enrichment Analysis

**Figure 4:**
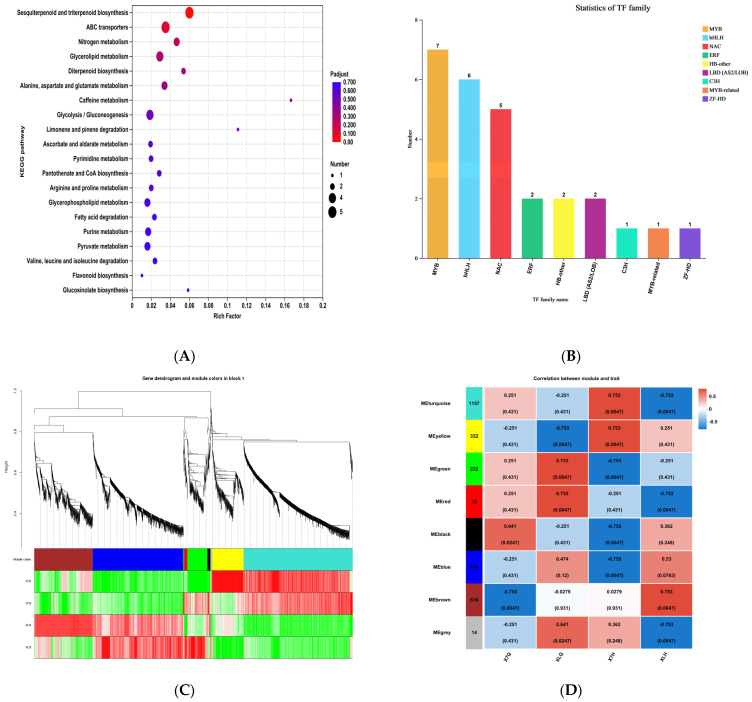
(**A**). KEGG enrichment analysis, (**B**). transcription factor family analysis, (**C**). differential gene cluster analysis, and (**D**). WGCNA modular identification.

A KEGG enrichment analysis was conducted on the shared differentially expressed genes (DEGs) identified in both the pre-abortion (X7_Q) vs. non-abortion (XL_Q) and post-abortion (X7_H) vs. non-abortion (XL_H) comparisons between ‘Xinli No.7’ and ‘Korla Fragrant Pear’. The results demonstrated that the DEGs were significantly enriched in the top twenty pathways (Figure 4A). Among these, six out of the twenty enriched pathways were associated with ROS-scavenging systems: galactose metabolism, ascorbate and aldarate metabolism, arginine and proline metabolism, fatty acid degradation, pyruvate metabolism, and flavonoid biosynthesis. The expression of these differentially expressed genes likely underlies the observed disparities in ROS content within the anthers of ‘Xinli No.7’ and ‘Korla Fragrant Pear’.

#### 2.3.4. Transcription Factor Analysis

Transcription factors (TFs) serve as central regulatory molecules in gene expression regulatory networks. Among the 283 DEGs identified in comparisons between ‘Xinli No.7’ and ‘Korla Fragrant Pear’, 27 transcription factor genes spanning 19 distinct families were characterized (Figure 4B). Analysis of family distribution characteristics revealed that the differentially expressed transcription factors were predominantly enriched in the MYB (seven members) and bHLH (six members) families. MYB and bHLH transcription factors, functioning as critical regulatory hubs in plant reproductive development, have been demonstrated to govern core processes, including anther tapetum development, dehiscence, and pollen maturation.

#### 2.3.5. WGCNA

Weighted gene co-expression network analysis (WGCNA) was conducted on 12 samples from ‘Xinli No.7’ and ‘Korla Fragrant Pear’, encompassing two developmental stages (triplicate biological replicates per cultivar-stage combination). Initially, genes exhibiting low expression variation (standard deviation ≤ 0.50) were filtered out, retaining 4599 genes for downstream analysis. Subsequently, a hierarchical clustering tree was constructed based on the dissimilarity coefficients between genes, and the dynamic tree cut algorithm was applied for module partitioning. Through calculation of module eigengenes and iterative merging optimization, eight gene co-expression modules were ultimately identified (Figure 4D). A correlational analysis between the eight modules and floral buds at different developmental stages of ‘Xinli No.7’ and ‘Korla Fragrant Pear’. In the late pollen abortion phase of ‘Xinli No.7’, highly significant positive correlations were observed with the MEturquoise and MEyellow modules, whereas strongly significant negative correlations were identified with the MEgreen, MEblack, and MEblue modules. Therefore, five modules exhibiting significant correlations with the late pollen abortion phase of ‘Xinli No.7’ were identified as key modules for subsequent in-depth investigation.

#### 2.3.6. ‘Xinli No.7’ Male Sterile Gene Mining

This study conducted an in-depth investigation into the anther development mechanisms of ‘Xinli No.7’. An initial functional annotation analysis was performed on the 283 DEGs identified through screening, integrating data from NCBI’s NR database, Universal Protein database, and peer-reviewed literature on anther development and male sterility. Through integrated KEGG pathway enrichment and weighted gene co-expression network analysis (WGCNA), we ultimately identified eight differentially expressed genes (DEGs) significantly associated with floral organ development, screened under stringent criteria (Table 2). Compared to the ‘Korla Fragrant Pear’, the male-sterile line of ‘Xinli No.7’ exhibited significant downregulation of seven genes (*Pbr012033.1*, *Pbr022331.1*, *Pbr035883.1*, *Pbr039379.1*, *Pbr040540.1*, *Pbr040761.1*, and *Pbr002037.1*) and significant upregulation of one gene (*Pbr015016.1*), as validated by RNA-Seq.

#### 2.3.7. qRT-PCR Validation

**Figure 5:**
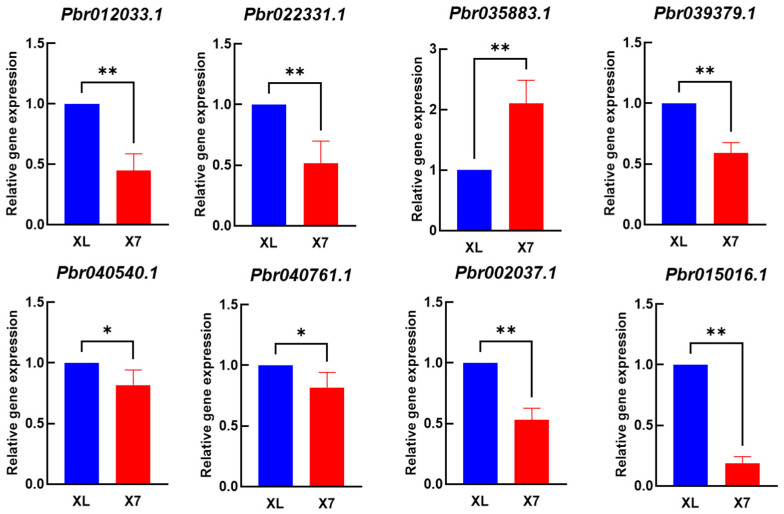
The expression levels of eight key differentially expressed genes in MF and MS plants were analyzed by qRT-PCR and RNA-Seq. The FPKM value is based on RNA-Seq data. The qRT-PCR was repeated three times, and the error bar indicated the standard error. The red stripe represents MS plants, and the blue stripe represents MF plants. ‘*’ indicated that the difference was statistically significant (*t*-test, *p* < 0.05), and ‘**’ indicated that the difference was extremely significant (*p* < 0.01).

A qPCR validation test was performed on these eight genes (Figure 5). qRT-PCR results demonstrated that all eight differentially expressed genes were expressed in both ‘Korla Fragrant Pear’ and ‘Xinli No.7’, with statistically significant differences in expression levels. With the exception of gene *Pbr035883.1*, which exhibited significantly higher expression in ‘Xinli No.7’ compared to ‘Korla Fragrant Pear’, all other genes demonstrated significantly or highly significantly lower expression levels in ‘Xinli No.7’. Among these eight genes, one may function as a positive regulator, while seven potentially act as negative regulators. This finding aligns with the expression trends identified through RNA-seq analysis.

## 3. Discussion

In most plant species, impaired programmed cell death (PCD) or structural abnormalities in tapetal cells constitute of the key factors leading to pollen abortion. As the nutrient ‘supplier’ for microspore development [21,22], the tapetum primarily functions to synthesize and transport enzymes, phytohormones, and nutrients essential for normal microspore development, thereby providing metabolic substrates critical for pollen maturation. This nutrient deprivation ultimately triggers microspore abortion. This study revealed that in ‘Xinli No.7’, the abortion of microspores is directly linked to vacuolation and abnormal proliferation of the tapetum. The aberrant differentiation process of the tapetal layer hinders the normal release of nutrients, directly triggering metabolic dysfunction in uninucleate pollen cells and causing their developmental arrest.

Proline plays multiple roles in plant floral development. Not only does it provide essential nutrients and energy for pollen development and pollen tube elongation [23,24], but it also functions as an osmoprotectant, regulating membrane permeability and modulating the activity of various enzymes [25]. Previous studies have demonstrated that the free proline content in floral buds of fertile plants is typically significantly higher than that in sterile plants during critical stages of microspore development, highlighting the vital role of proline in anther development [26]. Some studies suggest that anther abortion may be associated with impaired proline synthesis capacity [27]. The results of this study demonstrated that the proline content in ‘Xinli No.7’ was consistently lower than that in the fertile cultivar ‘Korla Fragrant Pear’ throughout anther development. Although both cultivars exhibited a sharp increase in proline content in early April, the magnitude of this increase was markedly greater in ‘Korla Fragrant Pear’ compared to ‘Xinli No.7’. These findings further corroborate the close link between proline content and male sterility in pears. Palif et al. [28] proposed that the free proline content within anthers is positively correlated with fertility and can serve as a key indicator of pollen fertility. Therefore, the relative reduction in proline content in ‘Xinli No.7’ may exert adverse effects on its fertility.

ROS play a dual role in plants. Optimal concentrations of ROS are essential for maintaining normal plant growth and development, whereas excessive accumulation can damage biological macromolecules such as lipids, proteins, and DNA, ultimately leading to cellular dysfunction [29]. The excessive accumulation of ROS has been recognized as a significant contributor to male sterility. Studies have shown that elevated ROS levels in anthers disrupt the temporal progression of tapetum degradation, while proper degradation of the tapetum is essential for pollen maturation [30,31,32,33,34]. This study found that the H_2_O_2_ content in ‘Xinli No.7’ was significantly higher than that in ‘Korla Fragrant Pear’ during most developmental stages, indicating that excessive accumulation of ROS in the anthers of ‘Xinli No.7’ may underlie the delayed degradation of its tapetum. In tomatoes, the *BZR1* gene participates in the generation of ROS. Mutations in this gene lead to delayed degradation of the tapetum and ultimately result in male sterility [35]. Similarly, excessive ROS concentrations in rice anthers also adversely affect pollen fertility [36,37,38].

This study revealed that the MDA content in ‘Xinli No.7’ remained consistently and significantly higher than that in ‘Korla Fragrant Pear’ throughout floral bud development, indicating a more severe degree of membrane system damage. Simultaneously, ‘Xinli No.7’ exhibited consistently lower activities of antioxidant enzymes such as SOD and CAT compared to ‘Korla Fragrant Pear’, while demonstrating significantly higher POD activity. This finding contrasts with research on male-sterile lines of *Vitis vinifera* ‘Shine Muscat’ [39], where POD activity was lower in sterile plants compared to fertile ones. The diversity in the regulation of enzyme activities may originate from interspecies variations in antioxidant defense mechanisms among different plant species. The critical physiological regulatory period underlying pear pollen abortion differs from the developmental stage at which male sterility occurs in grapes, resulting from the combined effects of external and intrinsic factors such as cultivation environment, genotypic background, and stress response mechanisms. This study revealed that both ‘Xinli No.7’ and ‘Korla Fragrant Pear’ exhibited an increasing trend in MDA content, a phenomenon that may be closely associated with the progressive decrease in SOD activity observed in both cultivars throughout the entire floral bud developmental stage. SOD, serving as the first line of defense in the antioxidant enzyme defense system of plant cells, often directly influences changes in MDA content through alterations in its activity [40].

Studies have demonstrated that seedless *Rosa roxburghii* exhibits lower SOD activity compared to common *Rosa roxburghii* while displaying significantly higher MDA content and POD activity [41]. Research has revealed that the male-sterile line of Festuca arundinacea exhibits lower SOD and CAT activity compared to the fertile line, whereas MDA content and POD activity consistently remain at higher levels [42]. The male-sterile line of watermelon exhibited significantly higher MDA content in floral buds compared to the fertile line, while demonstrating elevated POD and SOD activity [29]. Although most male-sterile lines exhibit MDA accumulation and elevated POD activity, their SOD and CAT activities display divergent trends. Some studies propose that reduced SOD and CAT activity may be critical factors contributing to male sterility [27], whereas the male-sterile lines of non-heading Chinese cabbage exhibit elevated protective enzyme activity. This phenomenon has been interpreted as an active defense response of the plant to membrane lipid peroxidation damage [29]. These results collectively demonstrate that the formation of the male sterility phenotype is closely linked to membrane lipid peroxidation damage triggered by ROS metabolic imbalance and the responsive mechanisms of the protective enzyme system.

This study aimed to investigate the genetic differences between ‘Xinli No.7’ and ‘Korla Fragrant Pear’. Transcriptome-sequencing technology was utilized to analyze floral buds at two developmental stages, identifying 4599, 4036, 3643, and 3788 DEGs, respectively. Fertility alterations in plants are typically accompanied by significant differences in transcript abundance, spatiotemporal expression patterns, and expression levels of associated genes. Therefore, systematic identification of these DEGs serves as a pivotal step for screening key fertility-regulating genes, laying the molecular groundwork for deciphering the regulatory networks governing reproductive development. Previous studies [43,44,45] have demonstrated that sterile anthers exhibit a significant enrichment of DEGs in the oxidative phosphorylation pathway and peroxidase pathway, where these DEGs play pivotal roles in regulating these pathways.

KEGG enrichment analysis revealed that six metabolic pathways—galactose metabolism, ascorbate and aldarate metabolism, arginine and proline metabolism, fatty acid degradation, pyruvate metabolism, and flavonoid biosynthesis—may be involved in ROS scavenging. Within these pathways, we identified four key genes: aldo-keto reductase family 4 member C10, alcohol dehydrogenase, aldehyde dehydrogenase family 3 member F1, and peroxidase 40. In *Arabidopsis thaliana*, Class III peroxidase 40 plays a crucial role in maintaining the integrity of the tapetal cell wall [46]. The prx9/prx40 double mutant exhibited abnormal morphological features, including swollen tapetal cells and cell walls intruding into adjacent cells, resulting in disrupted pollen exine deposition patterns and ultimately leading to the complete degradation of microspores. Furthermore, this study identified several genes, including chalcone synthase (CHS), ABC transporter G family member 9, transcription factor bHLH91, and ABC transporter G family member 15, which may play roles in the molecular mechanisms underlying male sterility in pear. ABCG transporters play a critical role in male fertility development in plants, primarily through regulating pollen wall development and anther cuticle formation. For instance, rice *OsABCG15*, serving as a functional homolog of Arabidopsis *AtABCG26*, plays a vital role during pollen development. Analysis of the pda1 mutant revealed that the absence of Ubisch body structures and impaired pollen exine formation led to abnormal microspore development and eventual abortion [47,48,49,50]. In maize, *ZmMS2*, a homolog of *OsABCG15* and *AtABCG26*, exhibits abnormal microspore outer wall development when mutated [49]. In the context of transcription factors, Yang et al. [51] identified three bHLH transcription factors (*AtbHLH10*, *AtbHLH89*, and *AtbHLH91*) and demonstrated that both double and triple mutants of these genes exhibit male sterility. Nakata et al. [52] identified and characterized three bHLH transcription factors *(JAM1*, *JAM2*, and *JAM3*) as key regulatory components of the jasmonic acid (JA) signaling pathway in Arabidopsis thaliana. Functional analysis revealed that overexpression of these transcription factors significantly inhibits stamen fertility, thereby demonstrating their negative regulatory roles during Arabidopsis stamen development. This discovery not only deepens our understanding of the crosstalk mechanisms between phytohormone signaling and reproductive development but also provides novel theoretical frameworks and potential molecular targets for deciphering the molecular regulatory network underlying male sterility in pear.

## 4. Materials and Methods

### 4.1. Experimental Materials

The experimental materials comprised ‘Xinli No.7’ and ‘Korla Fragrant Pear’ cultivated in the pear germplasm repository at Tarim University. The orchard soil was classified as sandy, grafted onto *Pyrus betulifolia* (Duli pear) rootstocks, with a planting spacing of 5 m × 4 m. Pear trees aged 26 years with open canopy structures and vigorous growth were selected as experimental materials, and anther samples were collected at different developmental stages.

### 4.2. Observation of Cell Development

From 1 March 2023 to 15 April 2024, floral buds (3–5 per collection) of ‘Xinli No.7’ and ‘Korla Fragrant Pear’ were sampled every seven days. The stamens were dissected, preserved in FAA fixative solution (formalin–acetic acid–alcohol), and vacuum-infiltrated to ensure tissue penetration. The conventional paraffin-sectioning method [53] was employed. Sections with a thickness of 6–8 μm were stained using the safranin-fast green counterstaining protocol, followed by dehydration, clearing, wax infiltration, embedding, sectioning, and mounting with resin. The samples were then observed and photographed using an Olympus BX251 light microscope. Based on the microscopic examination results, six sampling batches collected between March 8 and April 12 were selected for subsequent physiological index measurements.

### 4.3. Determination of Physiological Indicators

Samples stored at −80 °C were retrieved for proline content determination [54]. The levels of ascorbic acid (AA), hydrogen peroxide (H_2_O_2_), malondialdehyde (MDA), catalase (CAT), peroxidase (POD), and superoxide dismutase (SOD) were measured using assay kits purchased from Suzhou Comin Biotechnology Co., Ltd. First, 0.1 g of the sample was weighed and ground into powder in liquid nitrogen, and ascorbic acid (AA), hydrogen peroxide (H_2_O_2_), malondialdehyde (MDA), catalase (CAT), peroxidase (POD), and superoxide dismutase (SOD) were measured (Comin Jiangsu, China). All measurements were performed according to the manufacturer’s instructions, with three replicates per sample, and mean values were calculated. Column graphs were generated using GraphPad Prism (v. 8.0.2 for Windows), with results calculated from three biological replicates.

### 4.4. Transcriptome Sequencing

Anthers stored at −80 °C were transported on dry ice to Shanghai Majorbio Bio-pharm Technology Co., Ltd (Majorbio, Shanghai, China). for cDNA library construction and transcriptome sequencing. Total RNA was extracted from pear anthers using the RNeasy Mini Kit, and RNA purity and concentration were assessed through agarose gel electrophoresis and an ultramicro nucleic acid detector. The cDNA libraries were sequenced using the Illumina HiSeq2500 high-throughput sequencing platform [55].

### 4.5. Gene Differential Expression Analysis

Differential gene expression analysis was performed using EBSeq [56], identifying a set of DEGs between the two samples. Significant *p*-values were adjusted using the Benjamini–Hochberg method to control the false discovery rate (FDR). DEGs were screened using the criteria of fold change (FC) ≥ 2 and false discovery rate (FDR) < 0.05. The screened DEGs were subjected to GO functional enrichment analysis, KEGG pathway enrichment analysis [57], and WGCNA.

### 4.6. Validation of Candidate Genes Using Quantitative Real-Time PCR

A Plant RNA Extraction Kit (Omega, Beijing, China) was used to extract total RNA from the different anther developmental stages, and first-strand cDNA for the RT-qPCR was synthesized according to the instructions of HiScript II Q RT SuperMix (Vazyme, Nanjing, China). The total volume of the qPCR reaction system was 20 µL, including 10 µL of 2× ChamQ Universal SYBR qPCR Master Mix (Vazyme, Nanjing, China), each with 0.5 µmol/L of forward and reverse primers, 1 µL of ten-fold diluted cDNA template, and 8 µL of ddH2O. The PCR conditions were as follows: denaturation at 95 °C for 30 s; 40 cycles of denaturation at 95 °C for 10 s; and annealing and extension at 60 °C for 30 s. Gene expression was calculated using the 2^−∆∆Ct^ method. Detailed primer information is provided in Table 3, with *Pbr030912.1* serving as the reference gene. Three independent technical replicates were performed for each sample. *Pbr030912.1* was established as the reference gene, and relative gene expression levels were calculated using the 2^−ΔΔCt^ method.

## 5. Conclusions

This study has unveiled the integrated mechanism underlying male sterility in ‘Xinli No.7’ through a comparative analysis of the developmental processes leading to male sterility traits in ‘Xinli No.7’ and ‘Korla Fragrant Pear’. ‘Xinli No.7’ exhibited delayed anther dehiscence and a lack of pollen, characteristic of sterility. During the uninucleate microspore stage, swollen tapetal cells in ‘Xinli No.7’ compressed the microspores, resulting in morphological abnormalities and ultimately leading to microspore abortion. Conversely, in ‘Korla Fragrant Pear’, timely degradation of the tapetum enabled normal microspore development.

In contrast, during the uninucleate stage, the anthers of ‘Xinli No. 7’ exhibit excessive accumulation of ROS, significantly lower proline content compared to ‘Korla Fragrant Pear’, and markedly higher MDA levels, indicating severe membrane system damage. Meanwhile, ‘Xinli No. 7’ exhibited lower SOD and CAT activity compared to ‘Korla Fragrant Pear’, while demonstrating higher POD activity. This reduced capacity for ROS scavenging indicates that the process of microspore abortion is closely linked to membrane lipid peroxidation and dysregulation of the protective enzyme system.

Finally, the transcriptome analysis of anthers before and after the critical abortion stage (uninucleate stage) identified 4599, 4036, 3643, and 3788 DEGs, respectively. KEGG enrichment analysis revealed that six metabolic pathways—galactose metabolism, ascorbate and aldarate metabolism, arginine and proline metabolism, fatty acid degradation, pyruvate metabolism, and flavonoid biosynthesis—may participate in ROS scavenging. The transcription factor analysis of the differentially expressed genes (DEGs) revealed that a significant number of these genes belong to the MYB and bHLH families. Through a combined analysis of gene annotation and the literature review, eight candidate genes (*Pbr012033.1*, *Pbr022331.1*, *Pbr035883.1*, *Pbr039379.1*, *Pbr040540.1*, *Pbr040761.1*, *Pbr002037.1*, and *Pbr015016.1*) were screened and prioritized. The qPCR analysis revealed that among these eight genes, one positive regulatory gene (*Pbr035883.1*) and seven negative regulatory genes (*Pbr012033.1*, *Pbr022331.1*, *Pbr039379.1*, *Pbr040540.1*, *Pbr040761.1*, *Pbr002037.1*, and *Pbr015016.1*) were identified. This result is consistent with the expression trends observed in RNA-seq analysis.

## Figures and Tables

**Table 1:** Evaluation of sample sequencing data.

Sample	Raw Reads/Mb	Clean Reads/Mb	Q20 (%)	Q30 (%)	GC Content (%)	Total Mapped (%)
X7_Q1	55.73	55.44	98.99	96.75	48.29	75.65
X7_Q2	50.61	50.33	98.94	96.58	47.91	75.58
X7_Q3	53.07	52.75	98.96	96.67	47.75	75.25
XL_Q1	48.77	48.52	98.97	96.65	48.03	75.25
XL_Q2	51.20	50.92	98.96	96.65	48.14	76.26
XL_Q3	57.08	56.76	98.98	96.74	48.37	75.60
X7_H1	56.37	56.04	98.96	96.66	47.82	75.24
X7_H2	56.04	55.71	98.96	96.66	47.76	76.96
X7_H3	49.42	49.13	98.9	96.44	47.34	74.13
XL_H1	50.57	50.36	98.99	96.75	47.49	75.63
XL_H2	40.69	40.46	98.92	96.53	47.60	77.01
XL_H3	45.00	44.76	98.96	96.67	47.98	75.23

**Table 2:** Details of target gene expression.

Gene_id	Module	Regulate	X7H	XLH	Rate (XLH/X7H)	NR_Hit-Name
*Pbr012033.1*	green	down	1.096667	11.34667	10.3465	XP_009353802.1
*Pbr022331.1*	blue	down	0.86	88.17	102.5233	XP_009378509.2
*Pbr035883.1*	green	up	415.6033	184.61	0.4442	XP_009358773.2
*Pbr039379.1*	blue	down	7.143333	27.24	3.8133	XP_009360723.2
*Pbr040540.1*	yellow	down	2.25	10.65333	4.7348	XP_009346294.1
*Pbr040761.1*	brown	down	1.473333	20.89	14.1787	XP_009361467.2
*Pbr002037.1*	blue	down	0.326667	2.213333	6.7755	XP_009363200.2
*Pbr015016.1*	green	down	1.263333	12.81333	10.1425	XP_009372105.2

**Table 3:** Primers used in the RT-qPCR.

Gene_id	Forward Primer Sequence (5′-3′)	Reverse Primer Sequence (5′-3′)
*Pbr012033.1*	TGGCCTCATGATTTGCCTGT	CCGTAATGCCCACCAAGTCT
*Pbr022331.1*	GCTAGACGTCAACCCGCTTTTC	GGAGGAATGATGATTTTGGTGACGG
*Pbr035883.1*	TTCCTTTCTTGCCAACACCATCCC	TACGCCCGACCTGCTGACTTC
*Pbr039379.1*	GGAATGGTTTTGGTTTCTCCCG	ACCGGCAGTATCACACATTCTT
*Pbr040540.1*	CGTCACCGCCGCCATTAAG	CCGCTTCACTACACCATCATCG
*Pbr040761.1*	CCGCTTCACTACACCATCATCG	CCTCCGAGTGCGGTTAGGAG
*Pbr002037.1*	CTCCTTCAACAACACCACCACCTC	CCTCTGAGCCTTGCGAACTTCC
*Pbr015016.1*	CACTATTTCTGGGCGTTGCGA	AGGCTGCCATTCTTGGGTCT
*Pbr030912.1*	AGCCTTCCTGCCAACGAGT	TTGCTTCTCACCCTTGATGC

## Data Availability

Data are contained within the article. PRJNA1267957: Integrated cytological, physiological, and comparative transcriptome profiling analysis of the male sterility mechanism of Xinli No.7.
